# SARS-CoV-2 Seroprevalence in Western Romania, March to June 2021

**DOI:** 10.3390/medicina58010035

**Published:** 2021-12-26

**Authors:** Tudor Rares Olariu, Alina Cristiana Craciun, Daliborca Cristina Vlad, Victor Dumitrascu, Iosif Marincu, Maria Alina Lupu

**Affiliations:** 1 Discipline of Parasitology, Department of Infectious Disease, Victor Babes University of Medicine and Pharmacy, 300041 Timisoara, Romania; imarincu@umft.ro (I.M.); mariaalinalupu@gmail.com (M.A.L.); 2 Clinical Laboratory, Municipal Clinical Emergency Teaching Hospital, 300254 Timisoara, Romania; alinacristianacraciun@yahoo.com; 3 Center for Diagnosis and Study of Parasitic Diseases, Department of Infectious Disease, Victor Babes University of Medicine and Pharmacy, 300041 Timisoara, Romania; 4 Department of Biochemistry and Pharmacology, Victor Babes University of Medicine and Pharmacy, 300041 Timisoara, Romania; dalivlad@yahoo.com (D.C.V.); vicdumi@yahoo.com (V.D.); 5 Clinical Laboratory, County Clinical Emergency Teaching Hospital, 300723 Timisoara, Romania; 6 Clinical Laboratory, Institute of Cardiovascular Diseases, 300310 Timisoara, Romania

**Keywords:** SARS-CoV-2, antibodies, COVID-19, seroprevalence, epidemiology, Romania

## Abstract

*Background and Objectives:* The extent of SARS-CoV-2 infection among a population may be assessed by the presence of serum SARS-CoV-2 antibodies, which indicates previous exposure. The aim of this study was to determine the seroprevalence of SARS-CoV-2 infection in the adult population from Western Romania. *Materials and Methods:* Samples of 2443 consecutive individuals, referred for routine laboratory investigations, were tested for SARS-CoV-2 antibodies using the Elecsys immunoassay that targets the nucleocapsid protein, for identifying the presence of the total antibodies against SARS-CoV-2. *Results:* The overall SARS-CoV-2 seroprevalence was 45.60%. SARS-CoV-2 seroprevalence was significantly higher in age group 30–49 years (53.94%) compared to age groups 50–69 years (43.53%) and 70–91 years (30.79%) (*p* < 0.001, *p* < 0.001, respectively). No significant difference in seroprevalence was observed between females (44.83%) and males (47.05%). *Conclusions:* Our data revealed a high seroprevalence of SARS-CoV-2 infection in the adult population from Western Romania and indicate the rapid and significant spread of the virus. The estimated prevalence of 45.60% was 6 times higher than the rate of confirmed COVID-19 cases reported in the study area. This indicates the magnitude of virus transmission in the community.

## 1. Introduction

Since the first report of severe acute respiratory syndrome coronavirus 2 (SARS-CoV-2) in Wuhan, China, in late December 2019, the COVID-19 pandemic [1] has put the world’s healthcare systems under great pressure [2].

The prevalence of COVID-19 in different populations is still unclear and epidemiological surveillance of confirmed cases reflects only a part of all infected persons [3]. The true extent of the spread of the virus is underestimated [4] considering that the real number of asymptomatic SARS-CoV-2 infected persons is unknown [2].

Recent seroprevalence studies in Europe suggest that approximately one in five persons have been infected with SARS-CoV-2 after the second COVID-19 pandemic peak [5,6]. The extent of SARS-CoV-2 infection among a population may be assessed by the presence of serum SARS-CoV-2 antibodies [7] which indicate previous exposure [8].

Romania (19.4 million inhabitants) is among the most affected countries in Europe, with 1.796.230 SARS-CoV-2 reported cases and 58.019 deaths as of 17 December 2021 [9]. Timis County (705,113 inhabitants), located in Western Romania, reported 87.551 confirmed cases as of 17 December 2021 [10]. In Romania, on 26 February 2020, the first confirmed case of infection with SARS-CoV-2 was reported [11]. The state of emergency was declared on 16 March 2020, followed by the state of alert on 15 May 2020. Schools were closed on 11 March 2020 and remained closed until September 2020. Universities, both public and private, were also closed and all activities went online [12]. Vaccine eligibility opened to the adult general population on 15 March 2021. At that time, Pfizer-Biontech COVID-19 vaccine, Moderna COVID-19 vaccine, and AstraZeneca/Oxford COVID-19 vaccine were approved to be administered to the Romanian adult population.

In a study performed between July and September 2020, we reported a prevalence of 1.51% SARS-CoV-2 infection in Romanian blood donors [13]. However, the prevalence of SARS-CoV-2 infection in the Romanian adult population is currently unknown. In this study, we aimed to estimate the SARS-CoV-2 seroprevalence in the adult population from Timis County, Western Romania.

## 2. Materials and Methods

### 2.1. Study Design and Population

Between 10 March and 10 June 2021, we enrolled 2443 consecutive individuals from Timis County, Romania. Participants were included in the study in the order in which they presented for routine laboratory investigations to Municipal and County Clinical Emergency Teaching Hospitals Outpatient Clinics in Timisoara, Romania. Individuals at least 18 years old were included, with no upper age limit. Sampling was not based on online or website advertising, no social media/journals/TV or other public means were used for enrollment in this study. Participants were informed about the study only when they presented at the Municipal and County Hospitals Outpatient Clinics in Timisoara, for a routine check-up. All participants were informed about the purpose and the procedure of the study, including information regarding their laboratory investigations and interpretation of serologic test results. No specific inclusion or exclusion criteria were used.

Study participants were grouped as follows according to their age: 18–29 years, 30–49 years, 50–69 years, and 70–91 years.

### 2.2. Serologic Tests

Samples were tested for SARS-CoV-2 antibodies using the Elecsys Anti-SARS-CoV-2 immunoassay, designed for Cobas e analyzers (Roche Diagnostics GmbH, Mannheim, Germany), which uses a recombinant nucleocapsid protein (N) for identifying the presence of the total antibodies against SARS-CoV-2 (IgM, IgA, and IgG). The Elecsys^®^ is a double-antigen sandwich assay that uses the nucleocapsid protein for identifying specifically the presence of the total antibodies against SARS-CoV-2 infections, which are not generated after vaccination [14,15,16]. Antibodies to the nucleocapsid protein detect natural SARS-CoV-2 infection because this antigen is not targeted by the currently available vaccines in Europe [17]. The Elecsys^®^ has a specificity of 99.80% and a sensitivity of 99.5% for past infection in patients at ≥14 days after PCR confirmation. Interpretation of results was based on manufacturer’s criteria: samples with cutoff index ≥1.0 were considered positive. Quality control was performed according to the protocol specified by the manufacturer.

Sera were tested at the Clinical Laboratory of the Municipal Clinical Emergency Teaching Hospital in Timisoara, a reference laboratory for COVID 19 testing in Romania.

### 2.3. Statistical Analysis

Statistical analysis was conducted with Epi Info Version 7.2 (CDC, Atlanta, GA, USA) and Stata 16.2 (Statacorp, College Station, TX, USA). Chi-squared tests or Fischer’s exact test, as appropriate, were used to evaluate the differences between groups with respect to different characteristics. Logistic regression was used to examine the association between positive cases, areas of residence, gender, and age. Crude odds ratios and their 95% confidence intervals (95% CIs) were calculated. The logistic regression was conducted with Stata 16.1 (StataCorp, College Station, TX, USA). Statistical significance was set at *p* < 0.05.

This study was approved by the Ethics Committee of the Municipal Clinical Emergency Teaching Hospital in Timisoara, Romania. Written informed consent was obtained from those who accepted to participate in this study.

## 3. Results

Of the 2443 study participants aged 18–91 years (mean age = 52.44 ± 16.03 years), 65.29% (1595/2443) were females and 74.79% (1827/2443) were residents of the urban area (Table 1).

The overall seroprevalence of SARS-CoV-2 antibodies was 45.60% (1114/2443, 95%CI: 46.63–47.58). No significant difference in seroprevalence was observed between females (44.83%, 715/1595) and males (47.05%, 399/848) (OR = 0.91; 95%CI: 0.77–1.08; *p* = 0.305) (Table 1).

Overall, significantly higher seroprevalence of SARS-CoV-2 antibodies was identified in adults residing in rural areas (49.84%, 307/616) compared to those from urban areas (44.17%, 807/1827) (OR = 1.25; 95%CI: 1.04–1.51; *p* = 0.015). This difference was driven primarily by the significantly higher seroprevalence of SARS-CoV-2 antibodies in adults aged 30–49 years from rural areas (60.76%, 144/237) compared to urban areas (51.00%, 280/549) (OR = 1.48; 95%CI: 1.09–2.02; *p* = 0.012) (Table 1). Females from rural areas had higher prevalence of SARS-CoV-2 antibodies (50.79%, 193/380) compared to those from urban areas (42.96%, 522/1215) (OR = 1.37; 95%CI: 1.08–1.72; *p* = 0.007) (Table 1).

SARS-CoV-2 seroprevalence was significantly higher in age group 30–49 years (53.94%, 424/786) compared to age group 50–69 years (43.53%, 454/1043) (OR = 1.51; 95CI: 1.26–1.83; *p* < 0.001) and to age group 70–91 years (30.79%, 113/367) (OR = 2.63; 95%CI: 2.02–3.42; *p* < 0.001,) (Table 1).

The logistic regression analysis revealed that participants residing in rural areas were more likely to test positive compared to individuals residing in urban areas (OR = 1.25; 95%CI: 1.04–1.51; *p* = 0.015). Participants in the age group 70–91 years were less likely to test positive compared to individuals from other age groups (OR = 0.45; 95%CI: 0.32–0.63; *p* < 0.001). However, when the area of residence and age groups were combined in a logistic regression model, the area of residence was not found statistically significant (*p* = 0.075), but the age group 70–91 years remained statistically significant (*p* < 0.001).

## 4. Discussion

The SARS-CoV-2 seroprevalences in the general population reported in Europe after the second pandemic peak vary widely from, 4.06% in Slovenia [18] to 25.1% in Croatia [6]. Differences in sensitivity and specificity of assays used for testing [19,20], various sampling strategies with the study group [21], different sample sizes [22], or different times when studies were conducted [13] may contribute to the differences between the reported SARS-CoV-2 seroprevalences. Furthermore, the disparity in public health responses, local resources, population behavior [22], mitigation efforts, and the effectiveness of the implementation of prevention/control measures [20] may also explain the differences.

An increasing trend in the prevalence of SARS-CoV-2 infection during the second pandemic wave compared to the first COVID-19 pandemic wave was revealed by population-based SARS-CoV-2 seroprevalence surveys conducted in European countries. In France, for instance, the seroprevalence of SARS-CoV-2 antibodies raised from 2.1% [23] to 7.3% [24], and in Switzerland, the seroprevalence doubled since the end of the first wave reaching 21.1% [5]. In Southern Italy, between September 2020 and December 2020, the prevalence of SARS-CoV-2 infection almost tripled from 2.9% to 8.7% [25]. These data demonstrate the fast spread of the virus and may be explained by the milder measures implemented in the second pandemic wave compared to the first one [25], changes in policy and care-seeking behaviors, changes in test availability [5].

Our results indicate that almost 1 in 2 persons have been previously infected with SARS-CoV-2 at the end of the third COVID-19 pandemic wave. SARS-CoV-2 seroprevalence in Romania was higher than the rates recently reported in Switzerland (20.5%) [5] and Croatia (25.1%) [6] after the second COVID-19 pandemic peak, reflecting the extent and evolution of the pandemic during the third COVID-19 pandemic peak. The 45.60% seroprevalence of SARS-CoV-2 antibodies in the Romanian adult population was significantly higher compared to 1.51% SARS-CoV-2 seroprevalence in Romanian blood donors, reported in our survey performed between July and September 2020 [13]. Both studies were performed in Timis County, in the same laboratory, and with the same serologic assay. Despite the limitations of our blood donors study group, these results indicate a rapid and significant spread of the virus by the end of the third pandemic wave. 

The seroprevalence of 45.60% in our study group also demonstrates that the rate of SARS-CoV-2 infections is 6 times higher than the 7.28% rate of the confirmed cases in Timis County (51.382 cases of 705,113 inhabitants as of 12 April 2021, two weeks before the midpoint of sample collection). This also suggests that a significant proportion of asymptomatic or mild SARS-CoV-2 infections have been detected using serologic diagnostic methods.

As previously reported by other investigators [5,6,8,20,25], we did not observe a significant difference between females and males in our cohort.

Lower frequency of mask use in public spaces from rural areas compared to urban areas [26] may explain the significantly higher SARS-CoV-2 seroprevalence observed among adults from rural areas compared to those from urban areas in our study. In age group 30–49 years we observed a significantly higher seroprevalence of SARS-CoV-2 antibodies in adults residing in rural areas compared to those from urban areas and this is consistent with the results published by Paul et al. [27].

As previously shown, a higher SARS-CoV-2 seroprevalence was observed in adults aged 30–49 years and this may be explained by the fact that individuals from this age group are more involved in economic activities [28] and have many social activities with their peers [29]. Moreover, young adults are the main caregivers for children and parents in case they get infected with SARS-CoV-2 [29]. Children may be facilitators of SARS-CoV-2 transmission because, in most cases, infected children do not display notable symptoms [30]. It is still unknown whether schools are drivers for SARS-CoV-2 transmission, but it is likely that the increasing prevalence of infection in the community is related to school outbreaks [31].

Similar to other studies [6,32,33] we found SARS-CoV-2 seroprevalence to be lower in older individuals compared with younger adults. The elderly were probably less exposed to infection compared to young individuals and the working-age population.

In this study, participants were enrolled during the regular check-up for routine laboratory investigations and all collection centers were located in Timisoara city. This may have limited participation of some individuals from rural areas and may contribute to the lower number of study participants from rural areas compared to those from urban areas. In addition, antibody screening may fail to identify individuals in whom antibody levels decreased with time below the detection limit or those with low or weak antibody response. Previous studies indicated waning of antibody response within 4 months [34,35]. Although the Elecsys^®^ immunoassay is sensitive, it is possible that some convalescents from the first or second COVID-19 pandemic waves were not detected during our survey [35]. No epidemiological investigations or questionnaires regarding known past infection, presence of COVID-19 symptoms, contact with a confirmed case of COVID-19, vaccination status were conducted in this study. We cannot exclude that some of the study participants were contacts with a confirmed COVID-19 case or had a known past infection and this may be listed as a limitation. However, it is unlikely that the above limitations could significantly account for the seroprevalence noticed in our cohort. Therefore, we believe that the results of the present study suggest that SARS-CoV-2 seroprevalence in the adult population was high at the end of the third COVID-19 pandemic wave.

## 5. Conclusions

This study provides new and essential information regarding the seroprevalence of SARS-CoV-2 infection in the adult population and indicate the rapid and significant spread of the virus. The estimated prevalence of 45.60% was six times higher than the rate of confirmed COVID-19 cases reported in the study area. This indicates the magnitude of virus transmission in the community. In the light of a new pandemic wave, caused by the Delta variant (now dominant in much of the European Region) [36] and the decreasing trend of vaccination, health officials and policy makers should be aware of these results prior to implement new SARS-CoV-2 related policies.

## Figures and Tables

**Table 1:** Seroprevalence of SARS-CoV-2 among adults (*n* = 2443) in Western Romania according to age, gender, and area of residence.

	Males	Females	OR (95% CI)	*p*	Urban	Rural	OR (95% CI)	*p*	Overall	
Age Group (Years)	No. Positive/ No. Tested (%)	No. Positive/ No. Tested (%)			No. Positive/ No. Tested (%)	No. Positive/ No. Tested (%)			No. Positive/ No. Tested	% (95% CI)
18–29	33/78 (42.31)	90/169 (53.25)	0.64 (0.37–1.11)	0.132	89/180 (49.44)	34/67 (50.75)	1.05 (0.60–1.85)	0.886	123/247	49.80 (43.39–56.21)
30–49	151/257 (58.75)	273/529 (51.61)	0.74 (0.55–1.01)	0.067	280/549 (51.00)	144/237 (60.76)	1.48 (1.09–2.02)	0.012	424/786	53.94 (50.45–57.40)
50–69	174/359 (48.47)	280/684 (40.94)	0.73 (0.57–0.95)	0.021	346/800 (43.25)	108/243 (44.44)	1.05 (0.79–1.40)	0.767	454/1043	43.53 (40.55–46.56)
70–91	41/154 (26.62)	72/213 (33.80)	1.40 (0.89–2.22)	0.169	92/298 (30.87)	21/69 (30.43)	0.98 (0.55–1.73)	1.0	113/367	30.79 (26.29–35.69)
Total	399/848 (47.05)	715/1595 (44.83)	0.91 (0.77–1.08)	0.305	807/1827 (44.17)	307/616 (49.84)	1.25 (1.04–1.51)	0.015	1114/2443	45.60 (43.63–47.58)
